# A wound evaluation tool to prevent pressure ulcers

**DOI:** 10.3389/fsurg.2022.1037961

**Published:** 2022-11-08

**Authors:** Mingjian Zhao, Hongliang Zhao

**Affiliations:** ^1^ Graduate School, Dalian Medical University, Dalian, China; ^2^ Department of Burns and Plastic Surgery, Miyun Hospital, Capital Medical University, Beijing, China

**Keywords:** assessment, chronic wound, pressure ulcer, wound, prevention

## Introduction

Many elderly and patients with physical limitations are at risk of chronic pressure ulcers. There are many risk factors. For some, chronic pressure ulcers may affect the rest of the patient's lifetime (1). Serious pressure ulcers can become chronic wounds and the afflicted patient can die from sepsis or osteomyelitis due to complications from pressure ulcers. In the United States alone, pressure ulcers affect approximately 3 million adults annually. These adults have a diminished quality of life. The costs for the individual and healthcare system are high. Importantly, morbidity and mortality risks are significantly increased (2). Experts and doctors have many methods and tools to prevent pressure ulcers, including checklists and assessment methods (3, 4). These are highly professional and too complicated to be used by laypeople. The purpose of the article is to introduce a wound evaluation tool to be used by laypeople to communicate with doctors for the prevention of pressure ulcers.

## The structure of this tool

**Figure 1:**
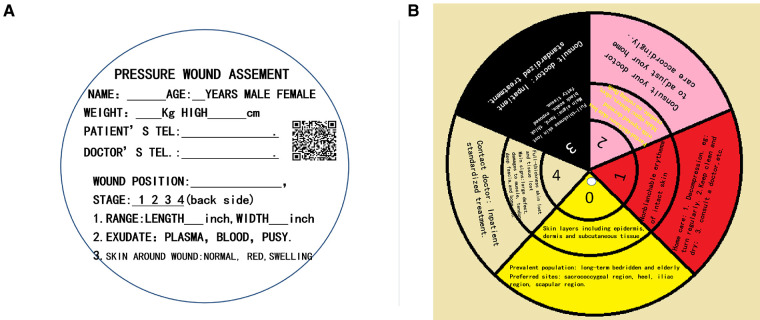
(**A**) The front of the big card: patients’ general information, wound evaluation, and WeChat QR code on the front side. (**B**) The back of the big card: a different color correlates to a different pressure ulcer stage. There are three circles that represent stage, skin injury, and relevant treatment, respectively.

This tool includes two round cards. These cards have identical round center holes and a snap fastener that keeps the two cards together. The card with the smaller diameter can rotate respectively. The card with the bigger diameter has two sides (front and back). General and wound information are printed on the front of the big card. The wound evaluation parameters include stage, area, exudate, and skin condition around wound. Only stages from 0 to 4 could be chosen based on the contents in the smaller card. The WeChat QR code of the hospital department or relevant institution is included with the patient's general information. The code takes the patient to a support group so that patients with this common problem can communicate with each other at any time. There are five color fan-shaped information areas on both cards. The big card also has three ring-shaped areas, while the smaller card has two ring-shaped areas. These three ring-shaped areas are successively distributed with basic ideas relating to the progression of the pressure ulcer and the relevant treatment (Figure 1).

## How to use this tool

This tool is designed to be read and understood easily within 10 min by laypeople. There are three steps. First, the patients or laypeople must be able to read and understand the information on the front side. This can be done easily during an education session. Second, several blanks must be filled in according to the patient's condition. Third, the wound evaluation parameters including stage, area, exudate, and skin condition around the wound must be considered. Only stages from 0 to 4 can be identified based on the information on the back of the big card. Only the small cards could be rotated. These three ring-shaped areas with five colors on the big card should be the same color as those on the small card.

## Discussion

The National Pressure Ulcer Advisory Panel (NPUAP) released the new terminology guidelines in 2016, The NPUAP's classification of pressure ulcer are graded from 1 to 6 (1). Grade 5 and 6 are too specialized and difficult for laypeople to consider and are best left to the doctors and experts. Grades 1–4 are easy to recognize and understand (Table 1). Therefore, we included grades 1–4 in this tool. Ideally, this tool should be presented to the patient and family as a part of pressure ulcer prevention at home, especially if the patient is inexperienced with pressure ulcers. The Norton, Waterlow, and Braden scales are classic scales for the assessment of pressure ulcers (1). The Norton scale score assesses five risk-based items, which range from 5 to 20 points, including physical condition, mental condition, activity, mobility, and continence. The Waterlow scale assesses eight items including body build (weight and height), visual evaluation of the skin, sex, age, continence, mobility, appetite, medication, and special risks (1). The Braden scale score assesses six risk-based items, which range from 6 to 23 points, including sensory perception, skin moisture, activity levels, mobility, observed nutritional intake, and friction and shearing forces (1). Although doctors and nurses take advantage of these scales in their work, these scales are too specialized and complicated for laypeople to use. Our wound evaluation tool has three important functions including wound evaluation, communication, and knowing the classification of the pressure ulcer and the relevant treatment. This tool could be read and understood within 10 min by literate laypeople. Hence, this tool is significantly different from the existing scales.

**Table 1:** The contents in three circles.

Grade	Skin injury	Relevant treatments
0	Skin layers including epidermis, dermis and subcutaneous tissue.	Prevalent population: long-term bedridden and elder. Preferred sites: sacrococcygeal region, heel, iliac region, scapular region, etc.
1	Nonblanchable erythema of intact skin.	Home care: 1. Decompression. For example: turn over regularly. 2. Keep clean and dry; 3. Consult a doctor, etc.
2	Partial-thickness skin loss with exposed dermis. Main signs: blisters, ulcer, exudate, no rotting flesh, etc.	Consult your doctor to adjust your home care accordingly.
3	Full-thickness skin lost. Main signs: hard, thick and black scabs, exposed fatty tissue, etc.	Consult doctors immediately: Inpatient standardized treatment.
4	Full-thickness skin lost and tissue lost. Main signs: large defect, damages to muscle, tendons, deep fascia, and bone.	Contact doctors immediately: Inpatient standardized treatment.

Two circles in smaller card including grade and skin injury. The rest one in big card including relevant treatments.

Communication between doctors and patients with pressure ulcer or their family members are usually difficult and ineffective. Patients with pressure ulcers usually also have a serious disease. Sometimes, the patients may not be able to tell others what is happening in detail. It is also very difficult for laypeople with no medical expertise to assess the pressure ulcer condition skillfully. Our tool aims to address these factors. The parameters included in this tool are easily read and understood. The multiple functions of this tool are designed to help the patient or their family member with timely wound assessment. That is to say, the important function of this tool is to help the patient or their family member to communicate with their doctor more easily and directly, especially in emergency situations. For example, the parameters such as stage, field, exudate, and skin condition around the wound, which could be read to the doctor or healthcare professional easily and directly. The descriptions are simple enough and based on the patient's pressure ulcer condition justly. The tool also includes contact phone numbers so that the patient can easily reach their doctor. The doctor/treating team can easily contact the patient for follow-up and provide additional assistance as necessary.

The tool also has another useful inclusion, namely, a QR code for the WeChat support group. Patients can join this support group and share any challenges relating to pressure ulcers on WeChat or seek assistance when they do not know what to do.

The tool is very instructive and useful for the prevention of pressure ulcers by laypeople. The earlier laypeople are made aware of pressure ulcers, the more effective the prevention of pressure ulcers. Moreover, this convenient tool can help the patient communicate more easily and directly with their doctor regarding their condition for the prevention of pressure ulcers. The obvious limitation of this tool is that it cannot replace expert medical advice. Doctors should not be replaced by such a tool. Consulting a doctor in time is essential to managing pressure ulcers.

In a word, this tool is significantly different from the classic scales. It may be understood and used by laypeople easily in order to prevent pressure ulcers.
